# Quetiapine and novel PDE10A inhibitors potentiate the anti-BuChE activity of donepezil

**DOI:** 10.1080/14756366.2020.1818739

**Published:** 2020-09-17

**Authors:** Joanna Sikora, Maria Podsiedlik, Tadeusz Pietras, Marcin Kosmalski, Mikołaj Matłoka, Rafał Moszczyński-Petkowski, Maciej Wieczorek, Magdalena Markowicz-Piasecka

**Affiliations:** a Laboratory of Bioanalysis, Department of Pharmaceutical Chemistry, Drug Analysis and Radiopharmacy, Medical University of Lodz, Lodz, Poland; b Department of Pharmaceutical Chemistry, Drug Analysis and Radiopharmacy, Medical University of Lodz, Lodz, Poland; c Department of Clinical Pharmacology, Medical University of Lodz, Lodz, Poland; d Research and Development Department, Celon Pharma S.A, Łomianki, Poland

**Keywords:** Alzheimer’s disease, acetylcholinesterase inhibitors, donepezil, PDE10A inhibitors, quetiapine

## Abstract

The symptoms of Alzheimer’s disease (AD) do not include only memory loss and cognitive decline but also neuropsychiatric manifestation. These AD-related symptoms are usually treated with the aid of antipsychotics; however, their effects on cognition and safety remain unexplored. The present study determines the effects of quetiapine, an atypical antipsychotic, and two imidazo[1,2-a]pyrimidine-based inhibitors of PDE10A on the activity of human cholinesterases. Quetiapine moderately inhibited BuChE (IC_50_ = 6.08 ± 1.64 µmol/L) but improved the anti-BuChE properties of donepezil by decreasing its IC_50_ value. Both PDE10A inhibitors were found to possess moderate anti-AChE properties. The combined mixtures of donepezil and imidazo[1,2-a]pyrimidine analogues produce a synergistic anti-BuChE effect which was greater than either compound alone, improving the IC_50_ value by approximately six times. These favourable interactions between quetiapine, PDE10A inhibitors and clinically approved donepezil, resulting in improved anti-BuChE activity, can lead to a wider variety of potent AD treatment options.

## Introduction

Alzheimer’s disease (AD) is an irreversible progressive neurological disorder. It is the most common cause for dementia and imposes immense suffering on patients and their families. AD is characterised by memory loss, the retardation of thinking and reasoning, and changes in personality and behaviours. It is estimated that there are currently about 46.8 million people suffer from AD worldwide. Furthermore, the number of patients is increasing, doubling every 20 years and is predicted to reach approximately 131.5 million in 2050^1^^,^^2^.

A comprehensive review of literature and clinical trials indicates that many hypothesis have been proposed regarding AD development: the cholinergic hypothesis, amyloid hypothesis, tau propagation hypothesis, oxidative stress and mitochondrial cascade hypothesis, calcium homeostasis hypothesis, neurovascular hypothesis, inflammatory hypothesis, metal ion hypothesis, and the lymphatic system hypothesis. It is well established that AD is a complex disease involving many factors, although its ultimate aetiology remains obscure. Although many attempts have been made to develop anti-AD drugs based on these hypotheses, only five drugs have been approved by the FDA to treat AD. Nowadays, the drugs used for AD treatments can be classified as either acetylcholinesterase inhibitors (AChEIs) or *N*-methyl-d-aspartic acid antagonists. Hence, there is a pressing need to develop more effective drugs for AD treatment^1–3^.

The process of developing new applications for a drug beyond its original use or commercially approved indication^3^, known as drug repurposing or reprofiling, is a relatively new idea. It is believed that such drug repurposing offers greater benefits over *de novo* drug discovery, i.e. the method of drug discovery by searching for a new active substance^4^^,^^5^. Moreover, it has gained increasing interest in recent years, mainly due to the fact that pharmaceutical companies seek competitive alternatives to compensate for the high costs and low success rate associated with the drug discovery process^4^. Repurposing also allows faster identification of new therapies for diseases, particularly in those cases where preclinical safety studies have already been accomplished^3^. Examples of successful repurposing can be found in existing literature. For instance, Baker et al.^3^ mention that bupropion, a drug originally used for depression, was repurposed for smoking cessation, while thalidomide, administered for treatment for morning sickness, is now used for multiple myeloma^3^.

Drugs have also been repurposed to treat neurodevelopmental and neurodegenerative disorders^6^. For instance, an extensive review by Bourque et al.^7^ examines the repurposing of sec steroids for the treatment of Parkinson’s disease (PD)^7^. In addition, fenfluramine, an appetite suppressant, can produce a durable reduction in seizures among patients with Dravet Syndrome, a rare genetic form of epilepsy^6^. Examples of repurposing of psychiatric drugs as anticancer agents are also given^8^.

Drug repurposing offers an opportunity to reinvigorate the drug discovery process for the treatment of AD^9–13^. Drug repurposing might become a promising alternative for AD treatment, since in the last few years, fewer than 25 potential drugs for AD have entered phase II and III clinical trials, compared to over 1700 anti-cancer molecules^10^.

Kumar et al.^12^ adopted a computational method based on ligand–protein interaction to explore potential antipsychotic drugs for the treatment of AD. The authors found that some antipsychotic drugs might exhibit encouraging potential against multiple targets associated with AD^12^.

Quetiapine is a psychotropic agent belonging to the group of dibenzothiazepine derivatives^14^. Quetiapine acts as an antagonist at multiple neurotransmitter receptors in the brain: serotonin 5HT1A and 5HT2, dopamine D1 and D2, histamine H1, and adrenergic α1 and α2 receptors^14^. The drug effectively alleviates positive and negative symptoms, as well as cognitive impairment in schizophrenia patients^14^. In addition to schizophrenia, quetiapine has been approved for the treatment of bipolar disorder and as add-on treatment of major depressive disorder^15^. The FDA extended the use of quetiapine to include generalised anxiety disorder, major depressive disorder, obsessive compulsive disorder, psychosis in PD, and treatment of behavioural and psychological symptoms in dementia, such as agitation, aggression, depression, and psychoses^16^. As presented by Schneider et al.^17^, quetiapine at a dose of 100 mg per day is effective for the treatment of psychotic symptoms and hostility in subjects with AD^17^^,^^18^. Takahashi et al.^19^ claim that quetiapine may be effective in treating psychotic symptoms and disruptive behaviour in some patients with dementia with Lewy bodies. There are also examples of *in vivo* animal studies evaluating the effects of quetiapine on various pathological hallmarks of AD^19^. For instance, He et al.^15^ report that quetiapine can alleviate cognitive impairment and pathological changes in an amyloid precursor protein/presenilin double transgenic mouse model of AD^15^. Furthermore, it has been claimed that quetiapine may improve cognitive symptoms of schizophrenia by stimulating brain-derived neurotrophic factor (BDNF) mRNA expression^20^. Hence, it is possible that quetiapine may serve as effective therapy in AD patients.

**Figure 1.**
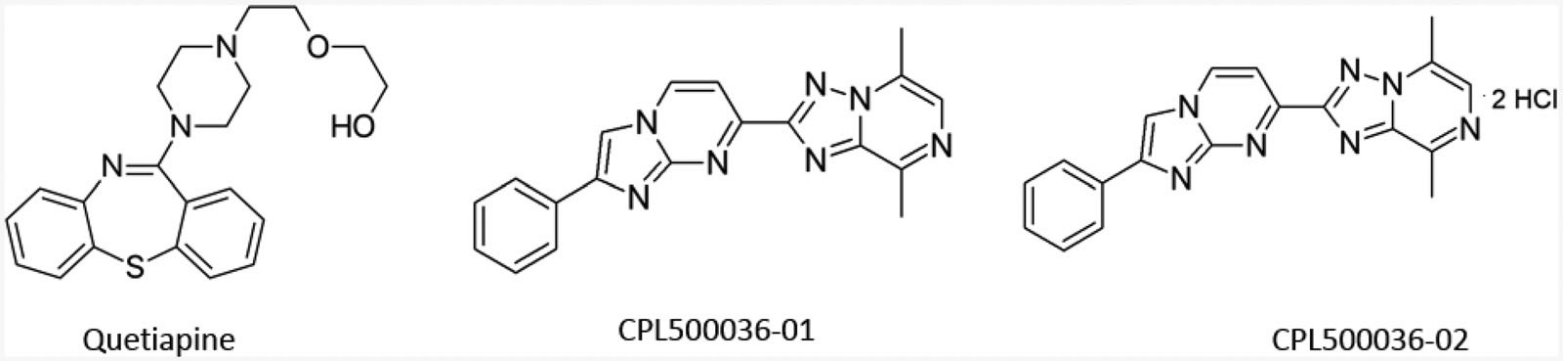
The chemical structure of quetiapine and PDE10A inhibitors, CPL500036-01 and CPL500036-02.

Therefore, the purpose of this *in vitro* study was to explore the effects of quetiapine and two novel antipsychotic compounds, currently undergoing clinical trials (Figure 1), on the activity of human acetylcholinesterase (AChE) and butyrylcholinesterase (BuChE), and to establish the type of inhibition. CPL500036-01 and its dihydrochloride salt (CPL500036-02) with imidazo[1,2-a]pyrimidine scaffold are new classes of PDE10A inhibitors characterised by high activity, good metabolic stability and satisfactory PK properties. In recent years, numerous studies of novel PDE10A inhibitors as potential antipsychotic agents have been published^21^.

The study also assesses the potential synergism of quetiapine, CPL500036-01, CPL500036-02, and donepezil towards both cholinesterases (ChEs). The findings provide a greater insight into the potential application of quetiapine as an effective adjuvant to clinically approved AChEIs.

## Materials and methods

### Materials

Quetiapine, CPL500036-01 and CPL500036-02 (which synthesis, affinity for PDE10A enzyme and metabolic stability were previously described^21^; Figure 1), were obtained from CelonPharma. Assuming the therapeutic plasma concentrations of quetiapine to be 4−400 ng/mL, i.e. 8−800 nmol/L (0.008−0.8 µmol/L), the compounds were examined within the concentration range 0.01−100 µmol/L.

The following reagents were used: 0.9% NaCl (0.15 mol/L; Chempur, Poland); 0.1 mol/L phosphate buffer pH = 7.0 and pH = 8.0 (disodium phosphate, monosodium phosphate [Baker, Poland]); a stock solution of 5,5′-dithiobisnitrobenzoic acid (DTNB; 0.01 mol/L [Sigma Aldrich, St. Louis, MO, USA]) prepared in phosphate buffer at pH = 7.0; a stock aqueous solution of acetyltiocholine iodide (ATC; 21.67 mg/mL; Sigma Aldrich); a stock aqueous solution of butyryltiocholine iodide (BTC; 20.50 mg/mL; Sigma Aldrich). The stock solutions of DTNB, ATC, and BTC were stored in low-volume aliquots at a temperature of −30 °C. Before each experiment the solutions were restored at 37 °C for 15 min. For the establishment of kinetic parameters and type of inhibition, decreasing concentrations of both substrates (ATC and BTC) were used (1:2 to 1:20).

### Biological material

The studies using biological material were approved by the Bioethics Committee of the Medical University of Lodz (RNN/109/16/KE – for compounds CPL500036-01 and CPL500036-02 analysis and RNN/278/19/KE – for quetiapine). Blood samples were obtained from healthy donors of Blood Donation Centre in Lodz and The Voievodal Specialised Hospital in Lodz. The preparation of erythrocytes for AChE activity measurements and plasma for the assessment of BuChE activity has been described previously^22^.

### Inhibition of ChE

The effects of quetiapine, CPL500036-01 and CPL500036-02 on the activity of both ChEs were determined according to Ellman with modifications^22^^,^^23^. Briefly, the experiments were conducted on a CE 2021 spectrophotometer (CECIL Cambridge, UK) with circulating thermostated water (37 °C) and a Model 300 Electronic Stirrer (Rank Brothers Ltd, England). The biological sample, consisting of a 400-fold diluted solution of haemolysed erythrocytes or 200-fold diluted plasma was incubated for 15 min (37 °C) with DTNB; the tested compound was then added in a volume of 10 µL. The enzymatic reaction was initiated by addition of a reaction substrate (ATC or BTC). The absorbance was continuously recorded at *λ* = 436 nm for three minutes, and the maximal velocity of the reaction was counted on the basis of changes in absorbance over time.

The method was validated, eight control tests were conducted both for AChE and BuChE. The coefficients of variability were counted (CV_AChE_ = 7.6%, CV_BuChE_ = 8.9%).

### Kinetic parameters of enzymatic reactions

To determine the type of BuChE inhibition by quetiapine, the experiments using decreasing concentrations (2-, 3-, 5-, 10-, 20-fold) of substrate (BTC) were conducted. The inhibitor was used at the IC_50_ value (6 µmol/L). A three-minute absorbance recording was carried out at *λ* = 436 nm using a CECIL 2021 spectrophotometer (CECIL Cambridge, UK) with a thermostatic water flow (temperature 37 °C).

Due to the high level of variation in the individual concentration and activity of BuChE in human plasma, 32 biological samples were studied. The following kinetic parameters were calculated for BuChE (mean ± SD; *n* = 32): *K*_m_ = 69.57 ± 21.34 µmol/L, *V*_max_ = 0.235 ± 0.027 A/min.

### ChE inhibition by binary mixtures of donepezil and tested compounds

The potential synergism between donepezil and quetiapine, CPL500036-01 or CPL500036-02 was estimated according to Ellman, with modifications^22^. Briefly, adequately diluted haemolysed erythrocytes or plasma (470 µL) were preincubated with donepezil (10 µL) and quetiapine or tested compounds (10 µL) for 15 min, before substrate addition (ATC or BTC at a final concentration of 0.75 µmol/mL). Donepezil was used at a concentration between 0.01 and 100 nmol/L for AChE inhibition, and 0.2 to 100 µmol/L for BuChE activity assessment. Quetiapine was administered at 200 ng/mL (400 nmol/L) while CPL500036-01 and CPL500036-02 were used at 25 µmol/L. The concentration of quetiapine was chosen on the basis of the mean concentration in plasma previously detected using HPLC^24^.

### Data analysis

All the experiments conducted within this article were carried out in duplicate or triplicate using at least three different biological samples. The values presented in tables and figures are expressed as mean ± standard deviation (SD).

The effects of quetiapine and compounds on ChE activity are presented as percentage of inhibition in comparison to controls, assumed to represent 100% of enzyme activity. The IC_50_ value, i.e. the concentration of a drug that inhibits 50% of the activity of an enzyme, was calculated using linear or logarithmic regression.

The kinetic parameters of enzymatic reactions were estimated using linear regression (Hanes-Woolf plots). The following parameters were calculated: the maximal velocity (*V*_max_) and the Michaelis constant (*K*_m_).

The effects of binary drug mixtures on the activity of ChEs were examined according to the median-effect principle described by Chou^25^ a method based on plotting dose-effect curves for every single drug and their binary mixtures in different doses. The potential synergism and antagonism between the drugs at all doses were thus analysed using commercially available ComboSyn software (http://www.combosyn.com/). The results of the analysis were obtained using a combination index (CI) plot. A CI value below 1 indicates synergism, while CI above 1 indicates antagonism between the examined drugs^26^.

## Results

### General ChE activity

**Figure 2.**
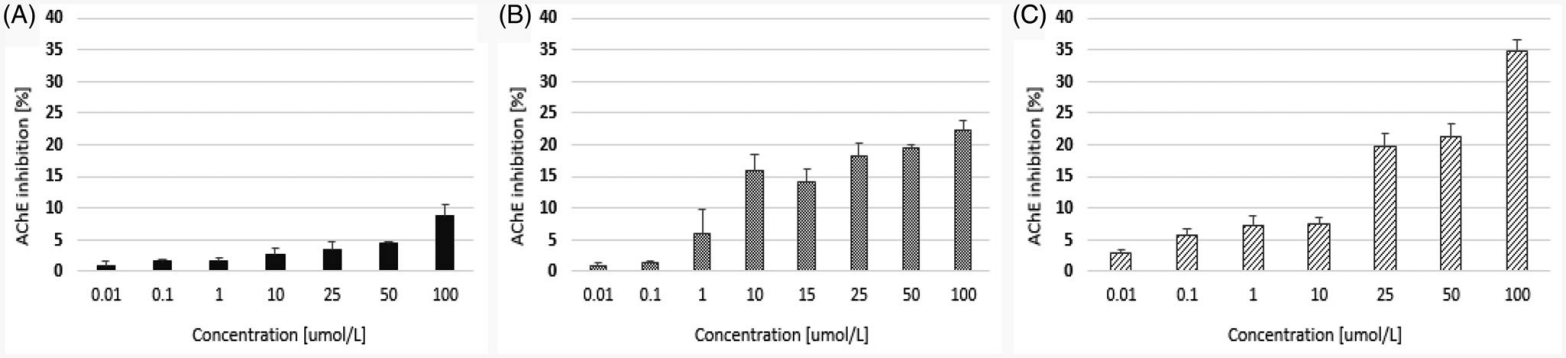
The effects of quetiapine (A) and CPL500036-01 (B) and CPL500036-02 (C) on AChE activity expressed as the percentage of enzyme inhibition in comparison to control (100% of activity). Each data point represents mean ± SD for at least three independent experiments conducted in duplicates.

**Figure 3.**
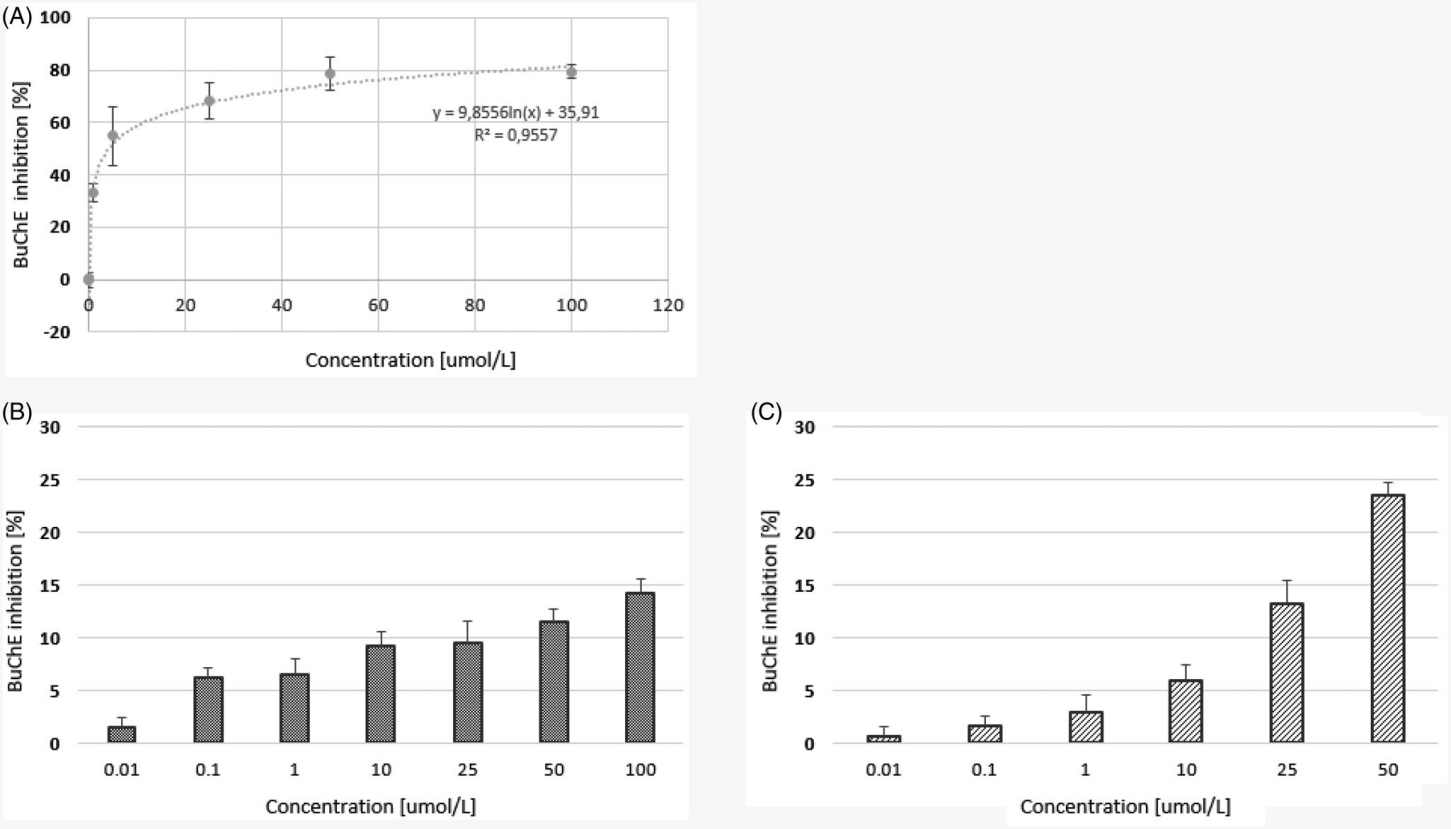
The effects of quetiapine (A) and CPL500036-01 (B) and CPL500036-02 (C) on BuChE activity expressed as the percentage of enzyme inhibition in comparison to control (100% of activity). Each data point represents mean ± SD for at least three independent experiments conducted in duplicates. Subsequent calculations using logarithmic equations from each conducted experiment allowed to determine the IC_50_ value for quetiapine.

The main objective of this study was to determine the effects of quetiapine and two novel compounds (CPL500036-01 and CPL500036-02) on the activity of human ChEs. Our findings (Figure 2(A)) indicate that quetiapine administered within the concentration range 0.01−100 µmol/L inhibited AChE inhibition by up to 8.82 ± 1.68%. In addition, the maximal inhibition towards AChE inhibition was 22.32 ± 1.46% for compound 1 and 34.87 ± 1.65% for compound 2 (Figure 3(B,C)).

Of the tested compounds, quetiapine presented the highest level of BuChE inhibition (IC_50_ = 6.08 ± 1.64 µmol/L). The two other compounds moderately inhibited BuChE activity within the tested concentration range (Figure 3(B,C)); however, the IC_50_ values could not be calculated. Compared to donepezil, a commercially approved drug for the treatment of AD quetiapine demonstrated approximately half the IC_50_ value against BuChE^22^, and compounds CPL500036-01 and CPL500036-02 were also significantly lower (Table 1).

**Table 1.** Effects of quetiapine and donepezil on the human erythrocyte acetylcholinesterase (AChE) and plasma butyrylcholinesterase (BuChE) activity.

Compound	IC_50_ (µmol/L)	
	AChE	BuChE
Quetiapine	NE	6.08 ± 1.64
Donepezil	0.025 ± 0.004	12.8 ± 1.52

The values are given as mean ± SD in three independent experiments conducted in duplicates on various biological samples. NE: non estimated (>100 µmol/L).

The value of CI below 1 gives evidence of synergism between the examined binary mixtures (don. + quet., don. + CPL500036-01, don. + CPL500036-02) in the case of BuChE inhibition. The results regarding AChE seem to be complex: higher concentrations mixtures also indicate synergistic activity.

### Kinetic parameters of BuChE enzymatic reactions

The kinetic parameters of BuChE reactions were obtained in a series of experiments using decreasing concentrations of BTC. The curves were analysed using the Hanes-Woolf equation, in which the ratio of the initial substrate concentration [S] to the reaction velocity *v* is plotted against [S].

**Figure 4.**
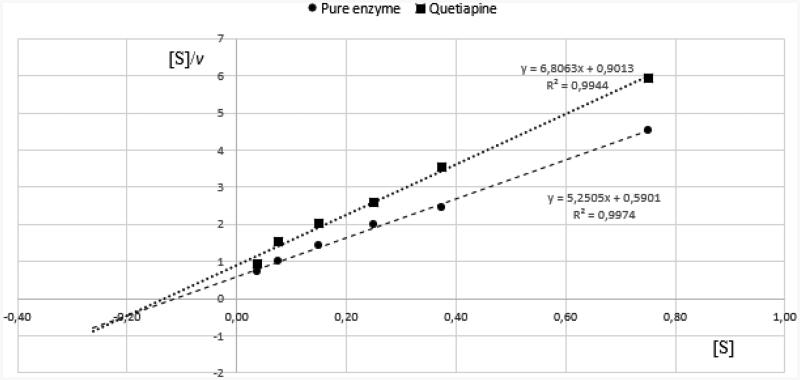
Determination of kinetic parameters of BuChE enzymatic reactions. Hanes-Woolf plots we used to calculate the Michaelis constant (*K*_m_) and maximal velocity (*V*_max_). Quetiapine was used at the concentration of 12.8 µmol/L. Presented data constitute the results of one exemplary experiment conducted in duplicates. The results of kinetic studies conducted in three independent experiments and calculated kinetic parameters are enclosed in Table 2.

The type of inhibition was determined on the basis of *K*_m_ and *V*_max_ values of the results obtained for pure enzyme and quetiapine at IC_50_ concentration (Table 2, Figure 4). According to the data presented in Table 2, quetiapine exhibited mixed inhibition, as *V*_max(i)_ (*V*_max_ of the reactions with inhibitor) significantly decreased in comparison with *V*_max_ while *K*_m(i)_ (*K*_m_ of the reaction with inhibitor) increased.

**Table 2.** Kinetic parameters of BuChE enzymatic reaction.

Drug	*K*_m_ (µmol/L)	*V*_max_ (A/min)	*K*_m(i)_ (µmol/L)	*V*_max(i)_ (A/min)
Quetiapine	92.7 ± 17.4	0.182 ± 0.041	103.5 ± 29.5	0.137 ± 0.036
Donepezil	85.3 ± 6.7	0.245 ± 0.069	149.7 ± 6.9	0.098 ± 0.032

The values are given as mean ± SD in three independent experiments.

### Inhibition of ChEs by donepezil and tested mixtures

In order to investigate the potential synergy between donepezil and quetiapine and the other two new compounds, several tests were performed using binary mixtures. The results of these experiments are presented in Table 3. Regarding BuChE activity, the greatest effect was demonstrated by the mixture of donepezil and compound CPL500036-01: IC_50_ was 2.13 ± 1.45 µmol/L, this value being about 83% lower than that of pure donepezil. In the case of 400 nmol/L quetiapine and 25 µmol/L compound CPL500036-02, the IC_50_ values decreased by 1.3- and 3.4-fold. Regarding AChE activity, similar IC_50_ values were obtained for a mixture of donepezil and quetiapine compared to pure donepezil. The mixture of donepezil with compound CPL500036-02 showed an IC_50_ of 21.72 ± 0.48 nmol/L, which was 15.09% lower than the IC_50_ of donepezil alone.

**Table 3.** Effects of the mixture of donepezil and quetiapine, or two new compounds on the human erythrocyte acetylcholinesterase (AChE) and plasma butyrylcholinesterase (BuChE) activity.

Compound	IC_50_	
	AChE (nmol/L)	BuChE (µmol/L)
Donepezil + Quetiapine	25.41 ± 1.82	9.82 ± 2.21
Donepezil + CPL500036-01	27.19 ± 2.66	2.13 ± 1.45
Donepezil + CPL500036-02	21.72 ± 0.48	3.78 ± 0.54
Donepezil	25.58 ± 4.56	12.81 ± 1.52

The values are given as mean ± SD in three independent experiments conducted in duplicates on various biological samples. Donepezil was used at the concentration between 0.01 and 100 nmol/L for AChE inhibition, and 0.2 to 100 µmol/L for BuChE activity assessment. Quetiapine, CPL500036-01, and CPL500036-02 were used at the following constant concentrations: 200 ng/mL (400 nmol/L) for quetiapine, 25 µmol/L for CPL500036-01, and CPL500036-02.

**Figure 5.**
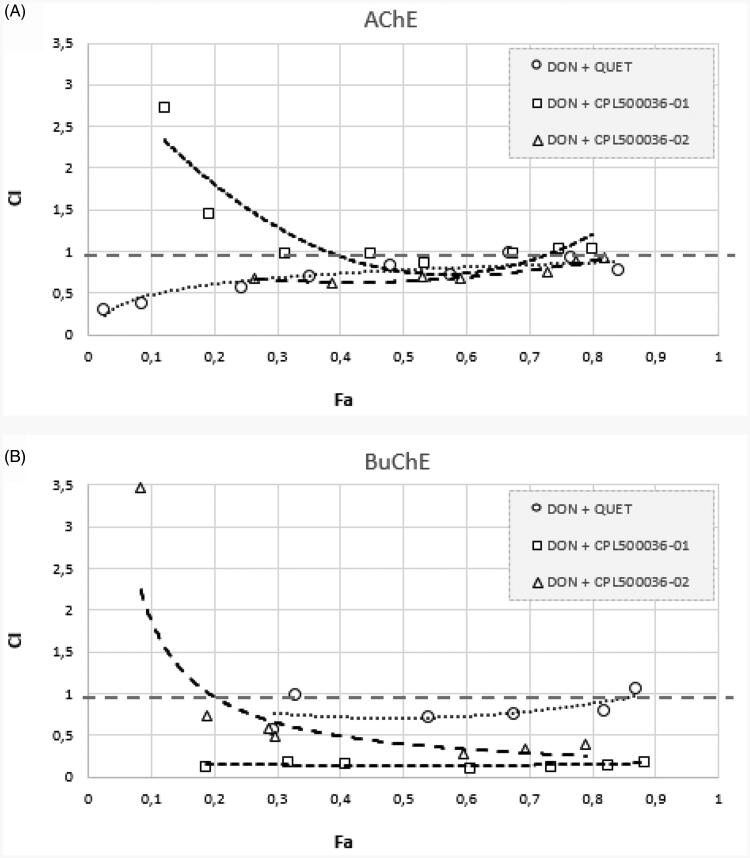
Analysis of potential synergism between donepezil and quetiapine, CPL500036-01, and CPL500036-02 by the median effect principle. Data from the AChE (A) and BuChE (B) inhibitory activities assay were analysed by means of the Chou–Talalay method. The results are depicted using Fa-CI plots, where Fa is Fraction affected, and CI is combination index.

The above results were confirmed using the median-effect principle (Figure 5). Figure 5(A) shows the anti-AChE activity of binary mixtures for which the CI value is in the range of 0.5–1, indicating synergy. The donepezil and CPL500036-01 mixture demonstrated CI values higher than 1 for the low Fa points, suggesting antagonistic dependency. Regarding the effects on BuChE inhibition (Figure 5(B)), all obtained Fa-CI curves are below 1, except for the donepezil and CPL500036-02 mixture at the smallest Fa, and the donepezil and quetiapine mixture at the highest Fa. The strongest anti-BuChE effect was demonstrated by donepezil and CPL500036-02 as shown by the position of the curve in the graph, i.e. close to CI = 0.

## Discussion

One of the most important challenges faced by contemporary science is the alleviation of neuropsychiatric symptoms associated with AD. As reviewed by Silva et al.^27^, some non-pharmacological strategies, such as music, aromatherapy, and pet therapy, have been applied to alleviate neuropsychiatric symptoms; however, their efficacy is disputable^27^. Regarding pharmacological interventions, a number of drug classes can be used: benzodiazepines, anticholinesterases, antidepressants such as selective serotonin re-uptake inhibitors (SSRI), mood stabilisers, anticonvulsants and antipsychotics^28^. For instance, SSRIs were found to be effective in the control of anxiety and depression; however, no significant difference was found between the efficacy of citalopram and risperidone for the treatment of either agitation or psychotic symptoms in subjects suffering from dementia. The authors conclude that these results need to be further confirmed before citalopram or other SSRI can be recommended as an option to antipsychotics for the treatment of agitation or psychotic symptoms associated with dementia^27^^,^^28^. In turn, Cummings et al.^29^ claim that results of numerous clinical trials of cholinesterase inhibitors and memantine confirm that the treatment offers behavioural benefits^29^.

The treatment of agitation and aggressiveness during the course of AD is usually carried out with the aid of antipsychotics^30^^,^^31^. According to McGrath^31^, up to 45% of patients in residential or nursing homes are treated with antipsychotics. Currently, interest is growing in the atypical antipsychotics, with over 20 clinical trials testing their efficacy^30^^,^^31^. For instance, a review of several clinical trials suggests that risperidone and olanzapine are effective in reducing aggression, and that risperidone reduces psychosis, but their administration is associated with serious adverse cerebrovascular events and extra-pyramidal symptoms. Therefore, the authors conclude that neither risperidone nor olanzapine should be used for a long period of time to treat dementia patients with aggression or psychosis^29–33^.

Although there is some clinical evidence that atypical antipsychotics exert moderate activity in the treatment of agitation and aggressiveness, a number of uncertainties still remain regarding their effects on cognition. An observational study of McShane et al.^32^ showed a doubling in the rate of cognitive decline in patients with dementia taking typical antipsychotics. Ballard et al.^33^ reported that administration of quetiapine does not result in significant improvement in agitation in patients with dementia compared with placebo and is associated with a greater decline in cognitive function. This has been attributed to the suppression of BDNF, accelerating the accumulation of the core pathological substrates of AD^33^, or to its antimuscarinic activity^34^^,^^35^. In contrast, Fleming et al.^36^ report that quetiapine offers potential cognitive benefits in people with schizophrenia. Lafuente and Thiamwong^37^ indicate quetiapine to be well-tolerated in patients with AD, and that it decreases clinical signs and symptoms of agitation; in addition, the use of quetiapine appears to be associated with cognitive stability^37^. De Deyn et al.^38^ report that treatment with quetiapine leads to improvement in the symptoms of psychosis and agitation, without any significant change in cognition^38^. Similarly, Roca et al.^39^ found that quetiapine administration in AD patients is not associated with any cognitive changes^39^.

Since one of the most important pathophysiological feature of AD is a deficit in cholinergic transmission, which can potentially influence all aspects of cognition, processing of information and behaviour, the present study evaluates the effects of quetiapine on the activity of two main human ChEs: AChE and BuChE. Most of the medicines currently registered for AD treatment act as AChEIs: they increase acetylcholine (ACh) levels in the synaptic cleft and partially ameliorate cognitive symptoms, and enhance quality of life in patients with mild to severe AD^40^.

Our present findings indicate that at 100 µmol/L, quetiapine exerts a maximal inhibitory effect of 8.82 ± 1.68% against AChE. This is the first report of the effects of quetiapine on the activity of human ChEs. This result is also of vital importance in view of the potential interactions between AChEIs and antipsychotic drugs. Since Mehrpouya et al.^41^ claim that co-prescription of AChEIs and dopamine D_2_ receptor blockers may induce an ACh/dopamine imbalance in the striatum, leading to extrapyramidal syndrome and extrapyramidal adverse effects, the present study examines the potential synergy between donepezil and quetiapine with regard to AChE inhibition. The findings (Table 3) indicate that quetiapine does not alter the AChE activity of donepezil, an approved drug for AD treatment. Therefore, we presume that the therapeutic potential of donepezil is not influenced by co-administration of quetiapine.

Another important target in AD therapy is BuChE. In contrast to AChE, which predominates in the healthy brain, BuChE is considered to play a minor role in controlling ACh levels. Notwithstanding, it has been found that BuChE level progressively increases in patients suffering from AD^42^. Therefore, both enzymes constitute potential therapeutic targets to alleviate a lack of ACh, one of the pathological hallmarks of the cognitive decline observed in AD. Our present findings indicate that against BuChE quetiapine demonstrated an IC_50_ value of 6.08 ± 1.64 µmol/L, which was roughly half that of the reference drug donepezil. However, it has to be noted that donepezil is an AChE-selective agent with approximately 500-fold greater affinity for AChE than BuChE.

A key finding of this study is that quetiapine supplements the anti-BuChE properties of donepezil, resulting in a 1.3-fold lower IC_50_ value. It is therefore vital to identify the potential synergism between recommended AChE inhibitors, such as donepezil, with antipsychotics which could lead to a wider variety of more potent treatment options in future.

The study also examines two novel compounds which primarily act by the inhibition of phosphodiesterase PDE10A. PDEs participate in many signalling processes, as they hydrolyse two crucial signalling molecules: cAMP and cGMP^43^. During the past 30 years, the issue of PDE distribution, substrate specificity, regulatory mechanisms and inhibition has constituted an active field of research. DeNinno^43^ describes the major advances in the field of PDEs which include identification of subtypes, and isoforms of PDEs, and understanding of their function^43^. Interest in PDEs has grown since the approval of moderately selective PDE5 inhibitors such as sildenafil, and tadalafil^43^^,^^44^. Numerous studies indicate that PDE inhibitors present a wide range of pharmacological activities, including anti-inflammatory, anti-oxidant, vasodilator, anti-cancer, and neuroprotective properties, suggesting that they can be used as potential drugs for the treatment of respiratory tract diseases, cardiovascular system diseases, depression, dementia, and PD^44–47^.

There is also great interest in the development of PDE inhibitors as potential anti-AD agents, since it has been found that AD is associated with the increased (CNS) expression of numerous PDE mRNA species^46^. In addition, inhibition of PDE5A decreases the level of phosphorylated Tau. Furthermore, inhibition of PDE10A was shown to have a positive regulatory effect on basal ganglia function, making it a potential target for the treatment of psychosis and schizophrenia^47^. The development of multitarget-directed ligand targeting ChEs and PDE is a promising approach to countering the multifactorial characteristics of AD^46^^,^^47^.

Our present findings show that both novel PDE10A inhibitors are characterised by greater potential for AChE inhibition than the reference drug, quetiapine, since its maximal inhibition by compound 1 and 2 reached approximately 22.32%, and 34.87%, respectively. The opposite situation was observed in the case of BuChE. In this case, the most effective compound was quetiapine (IC_50_ = 6.08 ± 1.64 µmol/L).

Our most significant result regarding the novel tested compounds is that they improved the anti-BuChE properties of donepezil: the IC_50_ value for the mixture of donepezil and CPL500036-01 was approximately sixfold lower than for donepezil alone (Table 3). The combination of donepezil and CPL500036-01, a PDE10A inhibitor, yielded a greater anti-BuChE effect than that for either compound alone. The observed synergistic effects might provide an additional rationale for any clinical benefit for patients suffering simultaneously from psychotic diseases and AD.

## Conclusions

It has been estimated that approximately 90% of demented patients, including those suffering from AD, develop neuropsychiatric symptoms such as delusion, aggressiveness and agitation^27^. The treatment of AD-related neuropsychiatric symptoms has been an area of extensive research in recent years. Some authors indicate that atypical antipsychotics exert moderate activity in the treatment of agitation and aggressiveness associated with AD, but their effects on cognition and ChE activity, the characteristic features of AD, remain unexplored. Therefore, our study examines the effects of quetiapine, an atypical antipsychotic, and two novel inhibitors of PDE10A^21^ on the activity of human ChEs.

Quetiapine was found to exert weak effects on AChE activity, but moderately inhibited BuChE activity (IC_50_ = 6.08 ± 1.64 µmol/L). Importantly, quetiapine improves anti-BuChE properties of donepezil, demonstrated by a 1.3-fold lower IC_50_ value. Although these findings suggest that possible beneficial interplay may exist between quetiapine and a clinically approved ChE inhibitor (donepezil), these preliminary results require further examination to rationalise the potential beneficial effects of combined therapy on the symptomatic treatment of AD with concomitant psychosis.

Both imidazo[1,2-a]pyrimidine analogues inhibiting PDE10A were found to possess greater anti-AChE properties than the reference drug, quetiapine; however, in contrast, their anti-BuChE effects were lower than those of quetiapine. The key finding of this article is the observation that both PDE10A inhibitors are capable of ameliorating the inhibitory properties of donepezil towards BuChE, which were manifested by ca. sixfold lower IC_50_ value.

Although anti-psychotics are still widely used for the treatment of the serious psychotic symptoms associated with AD, their use is associated with a number of uncertainties regarding their effects on cognition and possible adverse cerebrovascular events. Our findings reinforce the need to identify the potential synergism between recommended AChE inhibitors, such as donepezil, and antipsychotics; such knowledge could open the door to a wider variety of potent treatment options, or exclude adverse drug combinations resulting in impairments in ACh levels.
